# Age-Related Distribution and Severity of Coronary Artery Stenosis Assessed by 640-Row Coronary Computed Tomography Angiography: A Cross-Sectional Study

**DOI:** 10.7759/cureus.108970

**Published:** 2026-05-16

**Authors:** Dinh Nguyen, Son P Duong, Trieu V Tran, Hang Thanh T Nguyen, Tien N Tran, Vinh V Chu, Oanh Kieu T Nguyen

**Affiliations:** 1 Cardiology, Hoa Hao Can Tho Medic General Hospital, Can Tho, VNM; 2 Cardiology, Medic Medical Center, Ho Chi Minh, VNM; 3 Interventional Cardiology, Can Tho Central General Hospital, Can Tho, VNM

**Keywords:** 640-row ct, age factors, computed tomography angiography, coronary artery disease, coronary stenosis, vessel involvement

## Abstract

Background: Coronary computed tomography angiography (CCTA) is an established noninvasive imaging modality for evaluating suspected coronary artery disease (CAD). However, data describing the pattern of coronary stenosis and the extent of significant disease in patients undergoing modern 640-row CCTA remain limited.

Methods: This single-center cross-sectional study included 390 consecutive patients with suspected CAD who underwent 640-row CCTA between May 2021 and May 2022. Stenosis severity was assessed according to the coronary segment and major vessel involvement. Clinically significant stenosis was defined as luminal narrowing of ≥50% in the left main coronary artery (LM) or ≥70% in the left anterior descending, right coronary, or left circumflex arteries. The extent of significant CAD was categorized as single-vessel disease, two-vessel disease, or left main/three-vessel disease.

Results: The mean age of the study population was 58.4±10.4 years, and 209 patients (53.6%) were men. Women were older than men (61.4±10.3 vs. 55.8±10.1 years, p<0.001). Myocardial bridging was identified in 115 patients (29.5%) and was more common in men than in women (73/209 (34.9%) vs. 42/181 (23.2%), p=0.011). Across all coronary segments, most lesions were classified as none-to-moderate stenosis, whereas severe stenosis and total occlusion were less frequent. CCTA-detected significant stenosis was observed in 120 patients (30.8%), while 270 patients (69.2%) had non-significant stenosis. Single-vessel disease, two-vessel disease, and left main or three-vessel disease were present in 46 (11.8%), 41 (10.5%), and 33 patients (8.5%), respectively. Patients older than 60 years had significantly higher proportions of significant stenosis in the left main artery, left anterior descending artery (LAD), right coronary artery (RCA), and left circumflex artery (LCx) (all p<0.001). Among patients with significant CAD, the extent of disease differed by sex in the older age group but not in the younger age group.

Conclusions: In patients undergoing 640-row CCTA for suspected CAD, significant coronary stenosis was common and strongly associated with older age. Older men with significant CAD were more likely to have extensive disease, while myocardial bridging was more frequent and common in men. These findings highlight the clinical value of 640-row CCTA in the detailed characterization of coronary stenosis patterns in routine clinical practice.

## Introduction

Coronary artery disease (CAD) remains a major cause of morbidity and mortality worldwide, and the diagnostic evaluation of symptomatic patients with suspected chronic coronary syndrome has increasingly shifted toward structured risk assessment followed by selective noninvasive imaging. Contemporary European and North American guidelines place coronary computed tomography angiography (CCTA) at the center of this pathway because it can define coronary anatomy, identify or exclude obstructive disease, and support downstream preventive and therapeutic decision-making in stable chest pain [1-3].

The clinical value of CCTA is not limited to stenosis detection. In addition to its strong rule-out performance in appropriately selected patients, CCTA provides direct visualization of coronary atherosclerotic burden, plaque morphology, and anatomic variants such as myocardial bridging. Meta-analytic data show that the diagnostic performance of noninvasive tests varies across pre-test probability ranges, while major randomized trials such as Scottish Computed Tomography of the HEART (SCOT-HEART) and prospective multicenter imaging study for evaluation of chest pain (PROMISE) have demonstrated that a CCTA-based strategy can improve diagnostic certainty, guide therapy more precisely, and, in some settings, improve long-term outcomes [4-8]. Wide-detector systems with 320- and 640-row technology further strengthen this approach by enabling whole-heart coverage within a single heartbeat and supporting good image quality with reduced radiation exposure in selected protocols [9,10].

However, much of the published literature on wide-detector CCTA has focused on diagnostic accuracy, radiation performance, long-term outcomes, or detailed plaque analysis in large multicenter cohorts. In Vietnam, particularly in real-world clinical settings, evidence regarding 640-row CCTA remains limited. Existing Vietnamese reports have mainly addressed diagnostic correlation with invasive angiography or general coronary lesion characteristics rather than age- and sex-specific patterns of significant coronary stenosis detected by CCTA. Less is known about the real-world distribution of clinically CCTA-detected significant stenosis across individual major coronary vessels and how the extent of significant CAD varies by age and sex among Vietnamese patients undergoing 640-row CCTA for suspected CAD. This represents an important knowledge gap because age and sex are strongly associated with coronary atherosclerotic burden, yet vessel-specific stenosis patterns and patient-level disease extent may differ across populations, clinical settings, and imaging cohorts [11,12]. The specific focus on 640-row CCTA was based on its wide-detector capability for whole-heart coronary assessment in routine clinical practice, providing an appropriate platform for describing vessel-specific stenosis patterns in symptomatic patients. The primary objective of this study was to describe coronary stenosis characteristics detected on 640-row CCTA in a consecutive Vietnamese cohort of patients with suspected CAD, including stenosis severity by coronary segment and the frequency of significant stenosis in major coronary vessels. The secondary objective was to explore age- and sex-related patterns in the extent of significant coronary involvement detected by CCTA.

## Materials and methods

Study design and population

This was a prospective single-center cross-sectional diagnostic imaging study conducted at Hoa Hao Can Tho Medic General Hospital, Can Tho, Vietnam. Consecutive patients with suspected CAD who were referred for 640-row CCTA between May 2021 and May 2022 were screened for eligibility. The study was approved by the Department of Science and Technology of Can Tho and the Scientific Council of Hoa Hao Can Tho Medic General Hospital (Approval No. 56/QĐ-SKHCN). All participants agreed to participate after being informed about the indications, contraindications, and potential complications of the procedures. Personal information and medical records were coded and kept confidential.

The study population comprised patients aged ≥18 years presenting to Hoa Hao Can Tho Medic General Hospital, Can Tho, Vietnam, with suspected CAD, including those with stable chest pain and/or dyspnoea suggestive of chronic coronary syndrome, in whom obstructive CAD could not be excluded based on the initial clinical evaluation, basic investigations, or abnormal findings on prior non-invasive testing [13]. Patients were excluded if they had a history of iodinated contrast allergy, renal impairment with serum creatinine >150 μmol/L, coagulation disorders, acute medical conditions such as fever, infection, acute hepatic failure, or acute heart failure, or acute coronary syndrome.

The sample size was estimated using the single-proportion formula with a 95% confidence level and an absolute precision of 5%. Based on a previous 640-slice CCTA study by Di Cesare et al., which reported significant coronary stenosis in 32.5% of patients, the expected proportion was set at 0.325 [14]. The minimum required sample size was 337 patients. After accounting for approximately 10% incomplete or non-evaluable cases, the required sample size increased to 371 patients. Therefore, the final sample of 390 patients was considered adequate for the study.

Coronary computed tomography angiography acquisition

CCTA was performed using a 640-row scanner (Aquilion ONE; Toshiba Medical Systems, Tokyo, Japan). Patients were instructed to avoid coffee, tea, and other stimulants before scanning. If the heart rate was high or irregular, heart rate-control medication, including a beta-blocker, calcium-channel blocker, or ivabradine, was administered to reduce the heart rate to <70 beats/min. Patients were trained to hold their breath for 6-10 seconds before image acquisition. Scans were obtained with the patient in the supine position after ECG electrodes had been placed and a sufficiently large intravenous line had been established and connected to a dual-head power injector. Sublingual nitrate was administered before scanning to dilate the coronary arteries. The scan range extended from 1 cm below the tracheal bifurcation to the cardiac apex. Contrast volume was determined according to scan time and injection rate and typically ranged from 50 mL to 80 mL. Image reconstruction was performed automatically at the phases with the best image quality during systole and diastole, with additional manual reconstruction performed when needed. Image interpretation was performed by two experienced cardiovascular imaging physicians with prior experience in coronary CTA interpretation. The first 50 consecutive CCTA examinations were independently reviewed by both readers, who were blinded to each other’s assessments. Interobserver agreement for significant stenosis classification was almost perfect, with a Cohen’s kappa value of 0.85. Disagreements were resolved by consensus.

Definition of stenosis severity

For descriptive segment-level analyses, stenosis severity was categorized as no stenosis, mild stenosis (<50% luminal narrowing), moderate stenosis (50%-69%), severe stenosis (70%-99%), and total occlusion. Stenosis severity was assessed in the left main coronary artery (LM), left anterior descending artery (LAD), right coronary artery (RCA), and left circumflex artery (LCx).

For binary analyses, clinically significant stenosis was defined as luminal narrowing of ≥50% in the LM or ≥70% in the LAD, RCA, or LCx. For vessel-level analyses, a vessel was considered to have significant stenosis if its most severely affected segment met the corresponding criterion. Based on the presence of significant stenosis in each major coronary vessel, the extent of significant coronary involvement was further classified into three categories: single-vessel involvement, defined as significant stenosis in only one of the three major epicardial vessels excluding the LM; two-vessel involvement, defined as significant stenosis in any two major vessels excluding the LM; and left main or three-vessel involvement, defined as significant stenosis in the LM or in all three major vessels [15,16].

Statistical analysis

All statistical analyses were performed using R version 4.5.0 (R Foundation for Statistical Computing, Vienna, Austria). Data management and tabulation were conducted using the dplyr and tidyr packages. Categorical variables were summarized as frequencies and percentages, whereas continuous variables were presented as mean ± standard deviation (SD) or median with interquartile range (IQR), as appropriate. Comparisons of categorical variables, including stenosis severity and the extent of clinically significant coronary stenosis across age and sex groups, were performed using Pearson’s chi-square test, Fisher’s exact test, or the Fisher-Freeman-Halton exact test, as appropriate. Comparisons of continuous variables between two independent groups were performed using the independent-samples t-test. These analyses were implemented using the stats package. A two-sided p-value <0.05 was considered statistically significant.

## Results

Among the 390 enrolled patients, the mean age was 58.4±10.4 years, and 53.6% were men. Women were significantly older than men (61.4±10.3 vs. 55.8±10.1 years, p<0.001). Myocardial bridging was observed in 115 patients (29.5%) and was more common in men than in women (34.9% vs. 23.2%, p = 0.011). Across all evaluated coronary segments, most lesions were classified as none-to-moderate stenosis, whereas severe stenosis and total occlusion were less frequent. No significant sex differences were identified in the distribution of stenosis severity across the LAD, RCA, LCx, or LM segments (all p>0.05) (Table 1).

**Table 1:** Characteristics of the 390 patients ^a^Independent-samples t-test. ^b^Chi-square test. ^c^Fisher-Freeman-Halton exact test. LAD: left anterior descending artery; RCA: right coronary artery; LCx: left circumflex artery; LM: left main coronary artery

Characteristics		Male (n=209)	Female (n=181)	Total (n=390)	p	Test statictics
Age (year), mean ± SD		55.8±10.1	61.4±10.3	58.4±10.4	<0.001^a^	t=-5.41
Presence of myocardial bridging		73 (34.9)	42 (23.2)	115 (29.5)	0.011^b^	χ²=6.41
LAD1	None to moderate	188 (90.0)	152 (84.0)	340 (87.2)	0.095^c^	-
	Severe	21 (10.0)	29 (16.0)	50 (12.8)		
LAD2	None to moderate	171 (81.8)	142 (78.5)	313 (80.3)	0.702^c^	-
	Severe	36 (17.2)	37 (20.4)	73 (18.7)		
	Occlusion	2 (1.0)	2 (1.1)	4 (1.0)		
LAD3	None to moderate	194 (92.8)	166 (91.7)	360 (92.3)	0.698^c^	-
	Severe	14 (6.7)	15 (8.3)	29 (7.4)		
	Occlusion	1 (0.5)	0 (0.0)	1 (0.3)		
RCA1	None to moderate	194 (92.8)	175 (96.7)	369 (94.6)	0.152^c^	-
	Severe	13 (6.2)	4 (2.2)	17 (4.4)		
	Occlusion	2 (1.0)	2 (1.1)	4 (1.0)		
RCA2	None to moderate	182 (87.1)	166 (91.7)	348 (89.2)	0.184^c^	-
	Severe	25 (12.0)	15 (8.3)	40 (10.3)		
	Occlusion	2 (1.0)	0 (0.0)	2 (0.5)		
RCA3	None to moderate	188 (90.0)	173 (95.6)	361 (92.6)	0.122^c^	-
	Severe	18 (8.6)	7 (3.9)	25 (6.4)		
	Occlusion	3 (1.4)	1 (0.6)	4 (1.0)		
LCx1	None to moderate	198 (94.7)	167 (92.3)	365 (93.6)	0.456^c^	-
	Severe	9 (4.3)	13 (7.2)	22 (5.6)		
	Occlusion	2 (1.0)	1 (0.6)	3 (0.8)		
LCx2	None to moderate	183 (87.6)	164 (90.6)	347 (89.0)	0.316^c^	-
	Severe	21 (10.0)	16 (8.8)	37 (9.5)		
	Occlusion	5 (2.4)	1 (0.6)	6 (1.5)		
LCx3	None to moderate	191 (91.4)	173 (95.6)	364 (93.3)	0.124^c^	-
	Severe	14 (6.7)	8 (4.4)	22 (5.6)		
	Occlusion	4 (1.9)	0 (0.0)	4 (1.0)		
LM	None to mild	202 (96.7)	174 (96.1)	376 (96.4)	0.913^c^	-
	Moderate	4 (1.9)	5 (2.8)	9 (2.3)		
	Severe	3 (1.4)	2 (1.1)	5 (1.3)		

**Figure 1:**
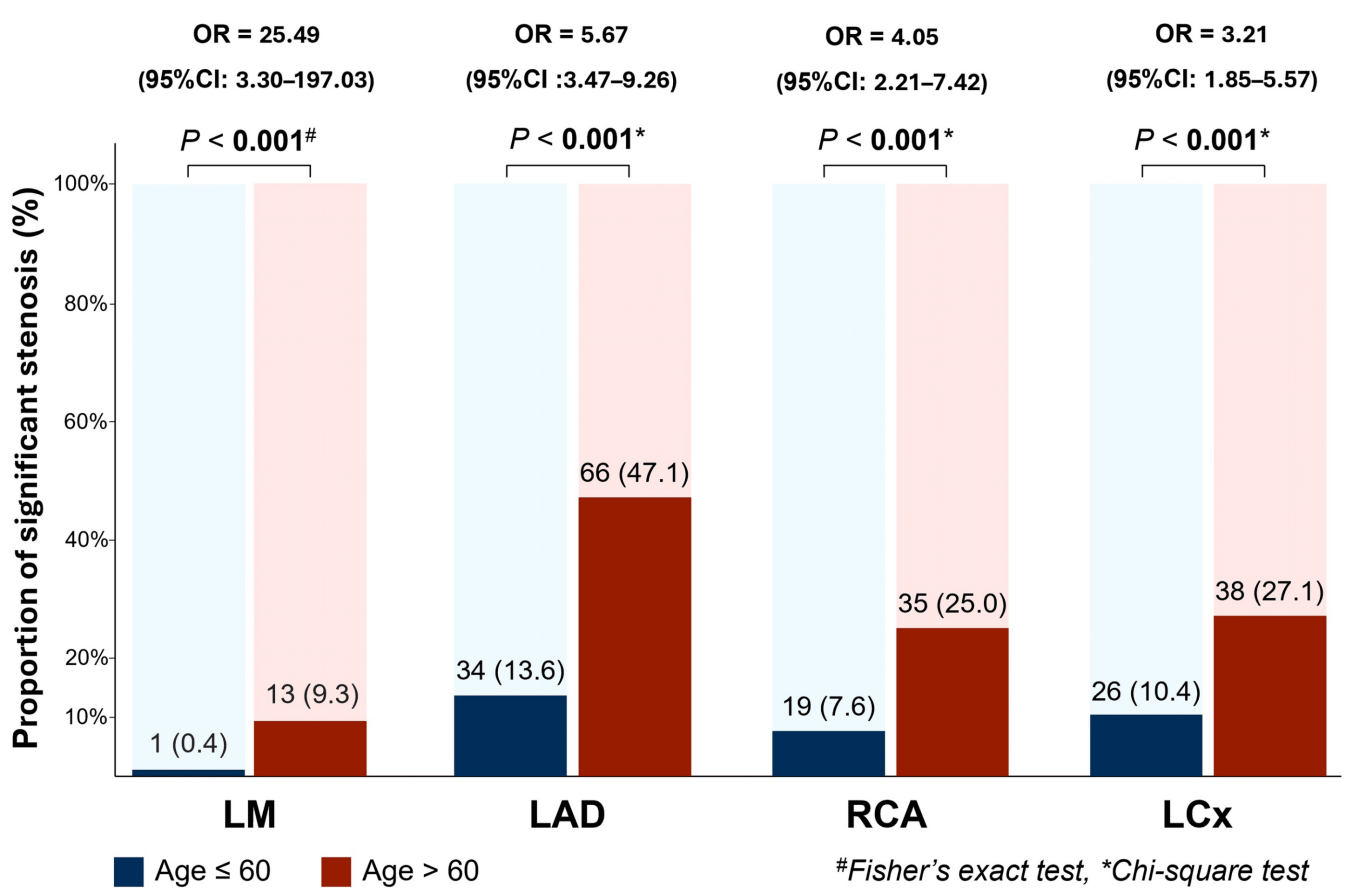
Proportion of significant coronary stenosis by vessel according to age group (n=390) LAD: left anterior descending artery; RCA: right coronary artery; LCx: left circumflex artery; LM: left main coronary artery; OR: odds ratio

Figure 1 shows that significant coronary stenosis was more frequent in patients aged >60 years than in those aged ≤60 years across all major coronary vessels. In the left main artery, significant stenosis was observed in 9.3% of patients aged >60 years and 0.4% of those aged ≤60 years (p<0.001). In the LAD, the corresponding proportions were 47.1% and 13.6% (p<0.001). In the RCA, significant stenosis was present in 25.0% versus 7.6% (p<0.001), whereas in the LCx the corresponding proportions were 27.1% and 10.4% (p<0.001).

**Figure 2:**
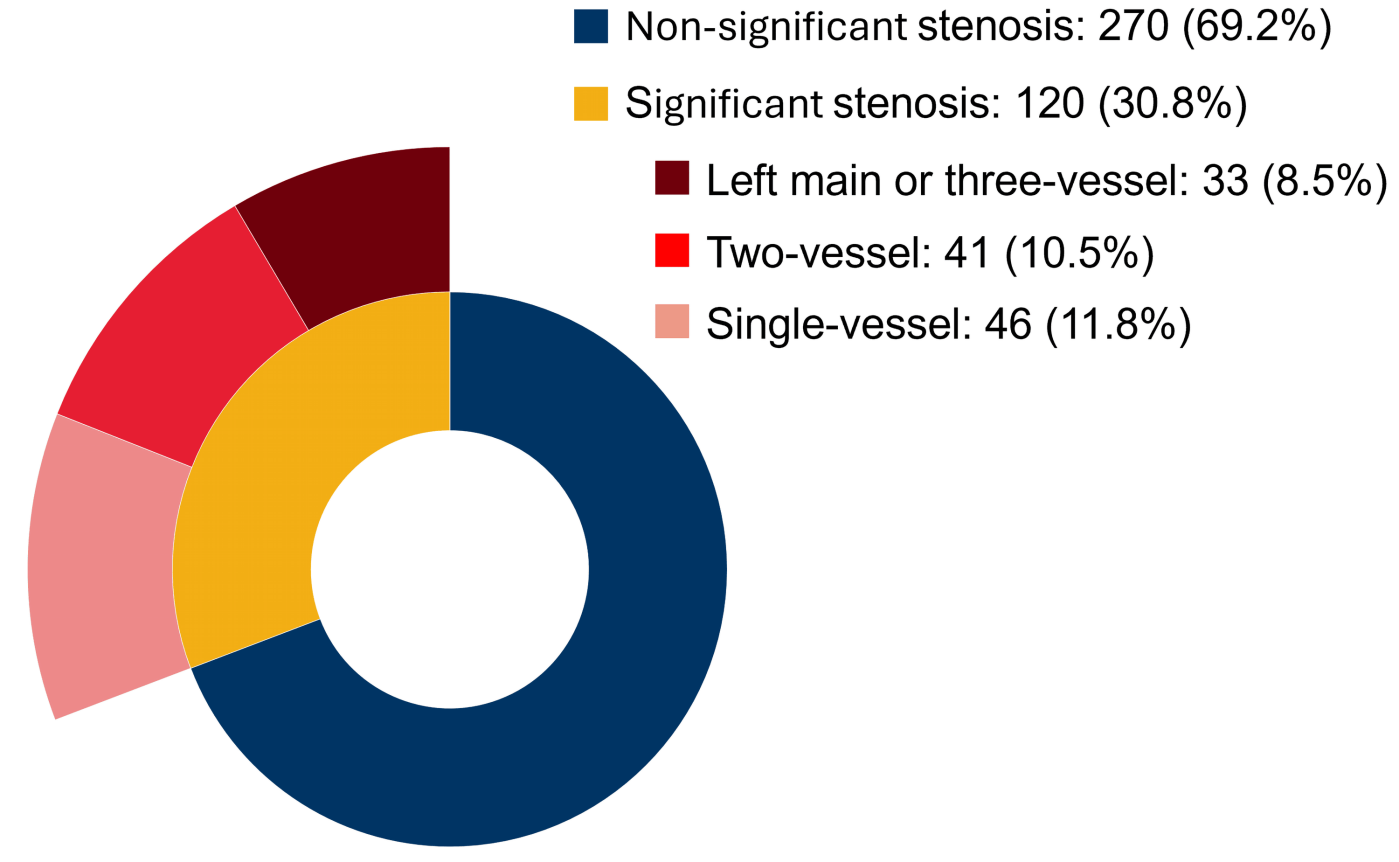
Distribution of significant CAD on CCTA (n=390) CCTA: coronary computed tomography angiography; CAD: coronary artery disease

As shown in Figure 2, 270 patients (69.2%) had non-significant coronary stenosis, whereas 120 patients (30.8%) had significant stenosis on CCTA. Among the overall study population, single-vessel disease was identified in 46 patients (11.8%), two-vessel disease in 41 patients (10.5%), and left main or three-vessel disease in 33 patients (8.5%).

**Figure 3:**
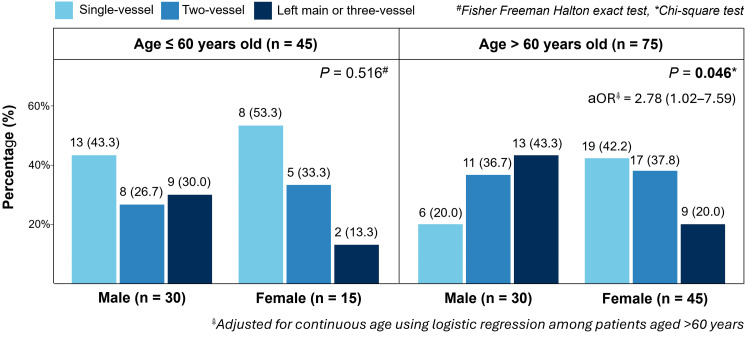
Extent of significant CAD by age and sex (n=120) ^#^Fisher-Freeman-Halton exact test. ^*^Chi-square test. CAD: coronary artery disease; aOR: adjusted odds ratio

Among patients aged ≤60 years, the distribution of single-vessel, two-vessel, and left main or three-vessel disease did not differ significantly between men and women (p=0.516). In men aged ≤60 years, the corresponding proportions were 43.3%, 26.7%, and 30.0%, respectively, whereas in women aged ≤60 years, they were 53.3%, 33.3%, and 13.3%, respectively. In contrast, among patients aged >60 years, the distribution differed significantly by sex (p=0.046). In men aged >60 years, single-vessel, two-vessel, and left main or three-vessel disease accounted for 20.0%, 36.7%, and 43.3%, respectively, compared with 42.2%, 37.8%, and 20.0%, respectively, in women aged >60 years (Figure 3).

## Discussion

In this single-center study of 390 consecutive patients with suspected CAD undergoing 640-row CCTA, most coronary segments showed no-to-moderate stenosis, whereas 30.8% of patients had significant CAD. Women were older than men, while myocardial bridging was more common in men. Significant stenosis was consistently more frequent in patients aged 60 years or older across the LM, LAD, RCA, and LCx. Among patients with CCTA-detected significant stenosis, the extent of disease differed by sex in the older group, with older men showing a higher observed proportion of left main or three-vessel involvement.

The age and sex pattern in our cohort is broadly in line with previous CCTA literature, although the exact balance differs across populations and disease definitions. In the PROMISE cohort of 10003 stable symptomatic outpatients, Hemal et al. found that women were older than men and were less likely to have a positive noninvasive test [17]. In a related PROMISE analysis, Pagidipati et al. showed that women were less likely to have a positive CTA than a positive stress test, whereas CTA conveyed strong prognostic information in women [11]. More recently, Williams et al. reported that women with stable chest pain had less plaque of all subtypes than men, and Yang et al., in a large Chinese multicenter cohort, showed that women developed coronary atherosclerosis later than men [18,19]. Taken together, these external data support the interpretation that age is a major driver of increasing plaque and stenosis burden, while sex differences may be more evident when plaque composition is measured quantitatively rather than when disease is classified only by luminal stenosis severity. Mechanistically, the age-related increase in CCTA-detected significant stenosis observed in our cohort is biologically plausible because vascular aging reflects cumulative exposure to inherited and acquired cardiovascular risk factors and is associated with endothelial dysfunction, arterial stiffness, and progressive atherosclerosis. These vascular changes can promote progressive plaque accumulation, calcification, and luminal narrowing with advancing age, which may explain why significant stenosis was more frequent in older patients in the present cohort [20]. Sex-related differences may also reflect differences in the timing and phenotype of coronary atherogenesis, as prior CCTA evidence suggests that women with stable chest pain generally have lower plaque burden across plaque subtypes than men [18].

Other CCTA data have also suggested that coronary plaque burden develops later in women than in men, supporting the possibility of delayed atherosclerotic progression in women [21]. Hormonal and vascular mechanisms may contribute to this pattern because estrogen has been described as having protective cardiovascular effects, while cardiovascular risk tends to increase after menopause [22,23]. Therefore, the higher observed proportion of left main or three-vessel involvement among older men in our cohort may reflect earlier and more cumulative atherosclerotic exposure in men, although this interpretation remains exploratory because baseline cardiovascular risk factors were not considered in the present analysis.

The proportion of patients with significant CAD in our study appears intermediate compared with large comparator cohorts, although direct comparisons require caution. Foldyna et al. reported an observed prevalence of only 13.9% for CAD ≥50% in the CTA arm of PROMISE, highlighting how contemporary North American chest pain cohorts often have lower obstructive disease prevalence than older probability models predict [24]. In contrast, Kim et al. in the invasive angiography-based Korean women’s chest pain registry (KoROSE) registry found a higher obstructive burden in men than women and more left main or three-vessel disease in men [12]. The Vietnamese context should also be considered when interpreting these differences. Cardiovascular disease is a major public health burden in Vietnam, accounting for 31% of all deaths in 2016 [25]. National stepwise approach to noncommunicable disease risk factor surveillance (STEPS) data also show a high and increasing burden of cardiometabolic risk factors, including raised blood pressure, raised cholesterol, overweight, insufficient fruit and vegetable intake, and highly sex-patterned smoking [26]. These population-level factors, together with local healthcare access and referral practices in southern Vietnam, may partly influence the spectrum of symptomatic patients referred for CCTA and the observed distribution of CCTA-detected stenosis in the present cohort. However, because baseline cardiovascular risk factors were not considered in the present analysis, this contextual interpretation remains hypothesis-generating. Therefore, our findings should be understood primarily as descriptive CCTA-based patterns of coronary stenosis in a Vietnamese referral population rather than as a risk-factor-based epidemiological estimate of CAD burden. Our myocardial bridging prevalence of 29.5% is higher than the 10% reported by Rajendran and Hegde in an Indian 64-slice CCTA series, but their study also emphasized that CT-based myocardial bridging prevalence ranges widely from 3.5% to 58% [27]. This wide variability is plausible and likely reflects differences in ethnicity, referral pattern, image quality, and acquisition technology. In that context, wide-detector CT is relevant because studies using 320- and 640-row systems have shown single-heartbeat whole-heart coverage, maintained diagnostic accuracy, and favorable radiation performance under optimized protocols [9,10].

This study has several strengths. It included a consecutive symptomatic population from routine clinical practice, used a standardized 640-row CCTA protocol, and assessed both segment-level stenosis severity and patient-level disease extent. The analysis also explored age and sex patterns, which are clinically relevant but not always reported in descriptive imaging studies. At the same time, several limitations should be acknowledged. First, this was a single-center cross-sectional study, so the findings may not be fully generalizable to other settings. Second, the study was based on CCTA findings and did not include a systematic comparison with an invasive or functional reference standard, such as invasive coronary angiography, fractional flow reserve, or ischemia testing. Therefore, the observed stenosis categories should be interpreted as CCTA-detected anatomical patterns rather than as confirmed anatomically or functionally significant CAD. Third, baseline cardiovascular risk factors, including hypertension, diabetes mellitus, dyslipidemia, smoking status, family history of CAD, BMI, and medication use, were not considered in the present analysis. Because these variables may confound age- and sex-based differences in coronary stenosis burden, the present comparisons should be interpreted as unadjusted descriptive comparisons of CCTA-detected stenosis patterns rather than evidence of independent associations. Fourth, radiation dose metrics, including CT dose index, dose-length product, and effective dose, were not analyzed. Therefore, this study cannot provide cohort-specific evidence regarding radiation exposure with the 640-row CCTA protocol, and the discussion of reduced radiation exposure should be understood as background evidence from prior optimized wide-detector CT protocols rather than as a finding of the present cohort. Fifth, the study did not include long-term clinical outcomes, so the prognostic significance of the observed stenosis patterns could not be assessed. Finally, some subgroup analyses were based on a limited number of patients, particularly after stratification by both age and sex.

Within these limitations, the present findings provide descriptive information on age- and sex-related patterns of CCTA-detected coronary stenosis in routine practice. In symptomatic patients undergoing 640-row CCTA, significant stenosis was more frequently observed in older patients, and older men with CCTA-detected significant stenosis showed a higher observed proportion of left main or three-vessel involvement. However, because the study did not include outcome follow-up, post-CCTA management data, or a comparison group, these findings should not be interpreted as evidence of prognostic value or treatment-guiding utility. Future research should move beyond luminal narrowing alone. Multicenter prospective studies incorporating standardized cardiovascular risk-factor data, quantitative plaque analysis, outcome follow-up, and comparison against invasive or functional reference standards are needed to clarify the clinical significance of 640-row CCTA findings in routine care.

## Conclusions

In conclusion, in this single-center descriptive cohort of patients with suspected CAD undergoing 640-row CCTA, most patients had no or non-significant stenosis, whereas nearly one-third had CCTA-detected significant stenosis. Significant stenosis was more frequently observed in patients older than 60 years across all major coronary vessels. Women presented at an older age, while older men with CCTA-detected significant stenosis showed a higher observed proportion of left main or three-vessel involvement. Myocardial bridging was frequent and was more common in men. Because this study did not include a reference standard, outcome follow-up, or a comparison group, these findings should be interpreted as descriptive and hypothesis-generating observations of CCTA-detected coronary stenosis patterns rather than as evidence of prognostic value, treatment-guiding utility, or superiority of 640-row CCTA over other imaging modalities.

## References

[1] Vrints C, Andreotti F, Koskinas KC (2024). 2024 ESC guidelines for the management of chronic coronary syndromes. Eur Heart J.

[2] Gulati M, Levy PD, Mukherjee D (2021). 2021 AHA/ACC/ASE/CHEST/SAEM/SCCT/SCMR guideline for the evaluation and diagnosis of chest pain: a report of the American College of Cardiology/American Heart Association Joint Committee on clinical practice guidelines. J Am Coll Cardiol.

[3] Narula J, Chandrashekhar Y, Ahmadi A (2021). SCCT 2021 expert consensus document on coronary computed tomographic angiography: a report of the Society of Cardiovascular Computed Tomography. J Cardiovasc Comput Tomogr.

[4] Knuuti J, Ballo H, Juarez-Orozco LE (2018). The performance of non-invasive tests to rule-in and rule-out significant coronary artery stenosis in patients with stable angina: a meta-analysis focused on post-test disease probability. Eur Heart J.

[5] SCOT-HEART investigators (2015). CT coronary angiography in patients with suspected angina due to coronary heart disease (SCOT-HEART): an open-label, parallel-group, multicentre trial. Lancet.

[6] Newby DE, Adamson PD, Berry C (2018). Coronary CT angiography and 5-year risk of myocardial infarction. N Engl J Med.

[7] Williams MC, Wereski R, Tuck C (2025). Coronary CT angiography-guided management of patients with stable chest pain: 10-year outcomes from the SCOT-HEART randomised controlled trial in Scotland. Lancet.

[8] Douglas PS, Hoffmann U, Patel MR (2015). Outcomes of anatomical versus functional testing for coronary artery disease. N Engl J Med.

[9] Di Cesare E, Gennarelli A, Di Sibio A, Felli V, Splendiani A, Gravina GL, Masciocchi C (2015). Image quality and radiation dose of single heartbeat 640-slice coronary CT angiography: a comparison between patients with chronic atrial fibrillation and subjects in normal sinus rhythm by propensity analysis. Eur J Radiol.

[10] de Graaf FR, Schuijf JD, van Velzen JE (2010). Diagnostic accuracy of 320-row multidetector computed tomography coronary angiography in the non-invasive evaluation of significant coronary artery disease. Eur Heart J.

[11] Pagidipati NJ, Hemal K, Coles A (2016). Sex differences in functional and CT angiography testing in patients with suspected coronary artery disease. J Am Coll Cardiol.

[12] Kim HL, Kim HJ, Kim M (2022). Sex differences in coronary angiographic findings in patients with stable chest pain: analysis of data from the KoRean wOmen'S chest pain rEgistry (KoROSE). Biol Sex Differ.

[13] Knuuti J, Wijns W, Saraste A (2020). 2019 ESC guidelines for the diagnosis and management of chronic coronary syndromes. Eur Heart J.

[14] Di Cesare E, Di Sibio A, Gennarelli A (2018). Low dose versus standard single heartbeat acquisition coronary computed tomography angiography. J Clin Imaging Sci.

[15] Cury RC, Leipsic J, Abbara S (2022). CAD-RADS™ 2.0 - 2022 coronary artery disease-reporting and data system: an expert consensus document of the Society of Cardiovascular Computed Tomography (SCCT), the American College of Cardiology (ACC), the American College of Radiology (ACR), and T. JACC Cardiovasc Imaging.

[16] Lawton JS, Tamis-Holland JE, Bangalore S (2022). 2021 ACC/AHA/SCAI guideline for coronary artery revascularization: executive summary: a report of the American College of Cardiology/American Heart Association Joint Committee on clinical practice guidelines. Circulation.

[17] Hemal K, Pagidipati NJ, Coles A (2016). Sex differences in demographics, risk factors, presentation, and noninvasive testing in stable outpatients with suspected coronary artery disease: insights from the PROMISE trial. JACC Cardiovasc Imaging.

[18] Williams MC, Kwiecinski J, Doris M (2021). Sex-specific computed tomography coronary plaque characterization and risk of myocardial infarction. JACC Cardiovasc Imaging.

[19] Yang X, Zhang J, Song Y (2025). Deciphering age- and sex-specific patterns of coronary artery atherosclerosis from a large Chinese cohort. Nature Communications.

[20] Herzog MJ, Müller P, Lechner K (2025). Arterial stiffness and vascular aging: mechanisms, prevention, and therapy. Signal Transduct Target Ther.

[21] van Rosendael SE, Bax AM, Lin FY (2023). Sex and age-specific interactions of coronary atherosclerotic plaque onset and prognosis from coronary computed tomography. Eur Heart J Cardiovasc Imaging.

[22] Xiang D, Liu Y, Zhou S, Zhou E, Wang Y (2021). Protective effects of estrogen on cardiovascular disease mediated by oxidative stress. Oxid Med Cell Longev.

[23] Ryczkowska K, Adach W, Janikowski K, Banach M, Bielecka-Dabrowa A (2023). Menopause and women's cardiovascular health: is it really an obvious relationship?. Arch Med Sci.

[24] Foldyna B, Udelson JE, Karády J (2019). Pretest probability for patients with suspected obstructive coronary artery disease: re-evaluating Diamond-Forrester for the contemporary era and clinical implications: insights from the PROMISE trial. Eur Heart J Cardiovasc Imaging.

[25] (2026). Cardiovascular diseases (CVD) in Viet Nam. https://www.who.int/vietnam/health-topics/cardiovascular-diseases.

[26] (2026). World Health Organization Regional Office for the Western Pacific: national survey on the risk factors of noncommunicable diseases in Viet Nam, 2021. https://www.who.int/publications/i/item/9789290620266.

[27] Rajendran R, Hegde M (2019). The prevalence of myocardial bridging on multidetector computed tomography and its relation to coronary plaques. Pol J Radiol.

